# A case report of lymphoma presenting initially with tracheoesophageal fistula and literature review

**DOI:** 10.1097/MD.0000000000046568

**Published:** 2025-12-12

**Authors:** Xunlai Lu, Luyao Ma, Li Ma, Yan Chen, Yuanjia Luo, Shengfu Liu, Wei Cao

**Affiliations:** a Department of Cardiovascular surgery, Northeast Yunnan Central Hospital, Zhaotong, China; b Department of Pathology, Northeast Yunnan Central Hospital, Zhaotong, China.

**Keywords:** chemotherapy, immunotherapy, Lymphoma, tracheoesophageal fistula

## Abstract

**Rationale:**

Extranodal involvement in non-Hodgkin lymphoma most commonly occurs in the gastrointestinal tract, whereas direct esophageal invasion by lymphoma is rare. The coexistence of a tracheoesophageal fistula (TEF) in such cases is even more exceptional, typically indicating a poor prognosis. This case report describes a female patient with advanced-age, large B-cell lymphoma involving both the lungs and esophagus, complicated by a TEF. Following treatment, the patient demonstrated improved physical strength and nutritional status, surviving more than 15 months post-diagnosis.

**Patient concerns:**

An 81-year-old female patient presented with a 2-month history of progressively worsening dysphagia, accompanied by cough and expectoration of white sticky sputum, along with marked weight loss and declining physical strength.

**Diagnoses:**

The patient was definitively diagnosed with extranodal metastasis of large B-cell lymphoma involving the esophagus and complicated by TEF combined imaging studies and endoscopic biopsy. Concurrently, the patient presented with multiple comorbidities, including pulmonary fungal infection, viral hepatitis, moderate aortic valve regurgitation, and atrial fibrillation.

**Interventions:**

The patient received 2 cycles of standard chemotherapy, concurrently supported by aggressive nutritional intervention via a jejunal feeding tube. This approach led to improved physical strength, stabilization of body mass index, and elevated serum albumin levels compared to baseline. A 1-year follow-up plain computed tomography scan demonstrated no further tumor progression.

**Outcomes:**

The patient's physical strength increased and nutritional status improved, with a post-diagnosis survival period exceeding 15 months.

**Lessons:**

This case underscores that individualized, effective symptomatic treatment can prolong survival and enhance quality of life in high-risk, frail oncology patients, while highlighting the critical role of nutritional support in comprehensive cancer care.

## 1. Introduction

Diffuse large B-cell lymphoma (DLBCL) represents the most prevalent subtype of non-Hodgkin lymphoma (NHL), accounting for approximately 40% of adult NHL cases in China, with the gastrointestinal tract being the most common extranodal site for lymphomatous involvement.^[1,2]^ Based on a review of the literature, it is exceedingly rare for TEF to present as the initial symptom in cases where the tumor concurrently involves both the lungs and esophagus.^[3]^ Patients often present with clinical symptoms such as dysphagia, chest pain, and weight loss, which typically warrant differentiation from primary esophageal malignancies. This report describes an elderly female patient who sought medical attention due to progressively worsening dysphagia. She was diagnosed with large B-cell lymphoma involving both the esophagus and lungs, complicated by a pretreatment TEF, an indicator of poor prognosis. Despite these challenges, a straightforward yet effective therapeutic approach achieved symptomatic improvement.

## 2. Case presentation

**Figure 1.**
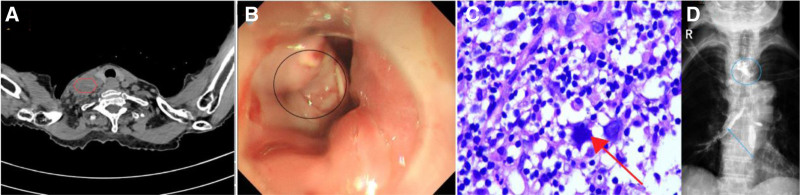
In Figure A, enlarged and fused lymph nodes (marked by the red circle) can be seen on the right side of the patient’s neck, with uneven density and a trend towards fusion. Figure B shows a concave mass on the lateral wall of the esophagus, with a ulcerated, uneven surface. A biopsy taken from this area confirms it to be a large B-cell lymphoma (indicated by the black circle). Figure C revealing atypical cells with prominent nucleoli occupying a significant proportion of the cell and coarse chromatin (indicated by the orange arrow; H&E staining, 40× magnification). Figure D is an anteroposterior view, showing contrast agent entering the trachea approximately 5 cm above the tracheal bifurcation (marked by the blue circle), and contrast agent entering the bronchus in the lower lobe of the right lung(marked by the blue arrow).

An 81-year-old female patient (height: 147 cm; weight: 37 kg) presented to our hospital on March 28, 2024, due to progressively worsening dysphagia over the preceding 2 months. Physical examination revealed a dyspneic appearance, cachexia, coarse breath sounds bilaterally on lung auscultation, irregular heart rhythm with unequal intensity of heart sounds, and no other significant positive findings. Upon admission, her BMI was 17.12, with laboratory results showing albumin 27 g/L, prealbumin 0.15 g/L, and total lymphocyte count 1.7 × 10⁹/L. Enhanced neck-thoracic CT demonstrated asymmetric thickening of the upper esophagus with mass formation, multiple enlarged lymph nodes in the bilateral cervical regions, right supraclavicular fossa, and mediastinum (suggestive of metastases) (Fig. 1A), and a 2.0 × 1.8 cm nodular density in the right lower lobe (suspected metastasis). Esophageal X-ray revealed a TEF located approximately 5.1 cm above the carinal bifurcation, with irregular mucosal contour at the corresponding esophageal segment (Fig. 1B). Esophageal endoscopy showed an ulcerated, irregular, depressed mass on the right lateral wall (15–23 cm from the incisors) (Fig. 1D). Endoscopic biopsy on April 2, 2024, confirmed diffuse large B-cell lymphoma (Fig. 1C). Additional evaluations revealed comorbidities including hepatitis B infection, fungal pneumonia, moderate aortic regurgitation, heart failure, and atrial fibrillation. Given her complex condition, the patient was diagnosed with DLBCL involving the lungs and esophagus, complicated by a tracheoesophageal fistula (AA stage: IVB), Eastern Cooperative Oncology Group Performance Status (ECOG) 3, cachexia, and multiple comorbidities. Surgical intervention was contraindicated. After multidisciplinary team (MDT) discussion, esophageal stent placement was deemed challenging, and the patient declined tracheal stenting due to family-related reasons.

On April 4, 2024, following comprehensive evaluation by the MDT, the patient initiated R + mini-CHOP chemotherapy (rituximab plus cyclophosphamide, doxorubicin, vincristine, and prednisone). Concurrently, she underwent endoscopic jejunal feeding tube placement after fasting. During hospitalization, enteral nutrition (via the jejunal tube) combined with intravenous support was administered to optimize nutritional status. The nutrition team formulated a tailored regimen, providing approximately 1200 kcal/day with 35% of energy from fat and 80 g/day of protein (primarily short-chain peptides and amino acids), supplemented with vitamins and trace elements. Comorbidities were managed symptomatically: entecavir was prescribed for hepatitis B antiviral therapy with continuous liver function monitoring; intravenous caspofungin was administered for fungal pneumonia, achieving sputum culture negativity after 2 weeks, followed by a 2-week oral consolidation course. Additional supportive measures included diuretics, potassium supplementation, and cardiac strengthening. At discharge on April 15, 2024, the patient demonstrated improved mental status and physical strength, with albumin increasing to 35 g/L, prealbumin to 0.19 g/L, total lymphocyte count to 2.0 × 10⁹/L, and ECOG performance status improving to 2. Outpatient enteral nutrition was continued, supplemented with low-residue liquid diets via the jejunal tube. The patient was readmitted on April 27, 2024, for the second chemotherapy cycle; however, 5 days post-treatment, she developed massive hematochezia. Endoscopy revealed a bleeding duodenal ulcer, and after endoscopic hemostasis failure, pancreaticoduodenal artery embolization was performed, resolving symptoms within 1 week (negative fecal occult blood). Despite clinical improvement, the family declined further antitumor therapy and requested symptomatic management only.

**Figure 2.**
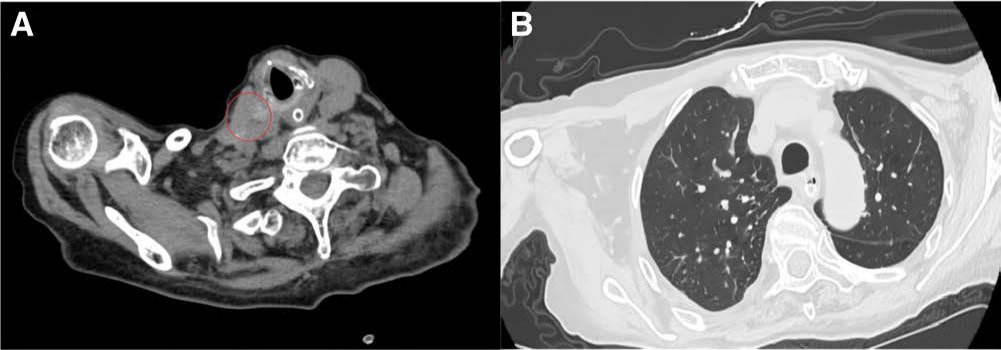
Figure A shows a significant reduction in the size of the previously enlarged and fused lymph nodes (marked by the red circle) observed 1 year ago in the patient. Figure B demonstrates the absence of significant aspiration pneumonia in both lungs 1 year later, suggesting adequate infection control.

Subsequent treatment excluded antitumor regimens. One month later, a follow-up gastroscopy was recommended, but the patient and family declined invasive procedures, posing a renewed challenge to therapeutic management. Symptomatic treatment alone was pursued, with sustained nutritional support playing a pivotal role. After 1 year, the patient’s weight increased to 41 kg, with albumin at 34 g/L, prealbumin at 0.18 g/L, and total lymphocyte count at 2.1 × 10⁹/L. In April 2025, a repeat chest CT demonstrated no significant tumor progression (Fig. 2), and the patient’s quality of life improved markedly over the past year. Despite advanced age and multiple comorbidities indicative of poor prognosis (e.g., TEF), the patient achieved tumor stabilization and comorbidity amelioration through 2 cycles of standardized chemotherapy and jejunal feeding tube placement for supportive care, surviving beyond 15 months. This case provides insights into managing DLBCL with extranodal thoracic involvement. The specific timeline refers to Table 1.

**Table 1:** Treatment schedule for patients with lymphoma

Time point	Event type	Details description
March 28, 2024	Consultation	The patient presented to our hospital with progressive dysphagia for 2 months. Physical examination revealed signs of dyspnea and cachexia.
At admission	Initial Assessment	BMI 17.12, albumin 27 g/L, prealbumin 0.15 g/L, total lymphocyte count 1.7 × 10^9^/L. Enhanced neck-thorax CT showed abnormalities in the esophagus and lungs.
April 2, 2024	Diagnosis	Esophageal endoscopic biopsy confirmed diffuse large B-cell lymphoma. Further evaluation revealed concurrent hepatitis B infection and fungal pneumonia.
April 4, 2024	Initiation of Chemotherapy	After multidisciplinary team assessment, the R + mini-CHOP chemotherapy regimen was initiated. Simultaneously, a jejunal feeding tube was placed for nutritional support.
April 15, 2024	Discharge	Discharged after chemotherapy, with improved mental status and physical strength. Albumin increased to 35 g/L, prealbumin to 0.19 g/L, total lymphocyte count to 2.0 × 10^9^/L, and ECOG score improved to 2.
April 27, 2024	Readmission for second chemotherapy cycle	The patient was readmitted for the second cycle of chemotherapy.
May 2, 2024	Complication	Five days after chemotherapy, the patient developed massive hematochezia. Endoscopy revealed duodenal ulcer bleeding. Endoscopic hemostasis failed, and pancreaticoduodenal artery embolization was performed.
May 9, 2024	Resolution of complication	Symptoms resolved after embolization, with negative fecal occult blood.
Mid-May 2024	Decision for supportive care	The family refused further anti-tumor treatment and requested only supportive care.
June 2024	Follow-up recommendation	Follow-up gastroscopy was recommended, but the patient and family declined invasive procedures.
June 2024 to April 2025	Ongoing nutritional support and follow-up	The patient continued to receive enteral nutritional support via a jejunal tube, supplemented with a low-residue liquid diet. Regular follow-ups were conducted to monitor the condition.
April 2025	Follow-up results	Weight increased to 41 kg, albumin 34 g/L, prealbumin 0.18 g/L, total lymphocyte count 2.1 × 10^9^/L. Repeat chest CT showed no significant tumor progression, and quality of life improved significantly.

This translated timeline table provides a comprehensive overview of the patient’s key events, including diagnosis, treatment, complication management, and follow-up results, facilitating a clear understanding of the patient’s disease progression and treatment journey.

## 3. Discussion

DLBCL is an aggressive malignancy characterized by significant heterogeneity in its biological behavior, clinical manifestations, and prognosis. The current treatment paradigm for DLBCL involves a multidisciplinary approach primarily centered on medical therapy. Notably, therapeutic strategies such as the R-mini-CHOP regimen, autologous hematopoietic stem cell transplantation, and chimeric antigen receptor T-cell (CAR-T) immunotherapy have substantially improved treatment outcomes for DLBCL patients.^[4–6]^ In particular, immune checkpoint inhibitors have emerged as a pivotal component in the treatment of multiple malignancies, demonstrating the potential for durable remission and offering promising prospects for long-term cure.^[7]^ For elderly (age > 80 years) frail patients with DLBCL and comorbidities, the R-mini-CHOP regimen is recommended as the preferred therapeutic option.^[8,9]^ This elderly patient presented with severe malnutrition, prompting the selection of the R-mini-CHOP regimen. The tumor exhibited high aggressiveness, with multiple extranodal metastases identified at initial diagnosis. After 2 cycles of chemotherapy, the patient discontinued subsequent antitumor therapy due to family-related factors, posing a challenge to treatment efficacy assessment. Ki-67, a critical biomarker for evaluating tumor proliferative capacity, malignancy grade, and prognostic outcomes,^[10]^ showed a value of 40% in this case, suggesting limited tumor proliferative activity. This may partially explain the stable disease status observed after 2 chemotherapy cycles. Combined with follow-up data, nutritional support therapy remained a key contributor to improved survival outcomes in this patient.

TEF demonstrates a relatively high incidence in malignant tumors such as esophageal cancer, lung cancer, and tracheal carcinoma, whereas secondary TEF arising from lymphoma is rare. When observed, it typically occurs following chemotherapy or radiotherapy.^[11]^ Malignant TEF is associated with an extremely poor prognosis, with a median survival time often not exceeding 3 months. Patients are prone to life-threatening secondary complications, including severe malnutrition, aspiration pneumonia, and massive hemorrhage.^[12]^ Acquired TEF predominantly occurs in adults, resulting from direct malignant tumor invasion or tissue necrosis secondary to chemoradiotherapy.^[13]^ Endoscopic and surgical interventions constitute the primary treatment modalities, with endoscopic approaches encompassing stent placement, titanium clip closure, and novel occlusive devices.^[14]^ In principle, surgical intervention should be considered as soon as possible following confirmation of TEF diagnosis.^[15]^ However, due to advanced age and cachexia, this patient was deemed ineligible for surgical intervention. In the context of conservative management, antimicrobial therapy and nutritional support emerged as critical for stabilizing the patient’s condition. The family’s refusal to consider tracheal side-stent placement posed challenges in timely fistula occlusion, which complicated pulmonary infection control. Notably, adequate antifungal therapy demonstrated excellent efficacy against aspiration-related fungal pneumonia. Early diagnosis and prompt treatment initiation were pivotal to successful infection management. Aggressive enteral nutrition via jejunal feeding tube, addressing basal energy requirements with optimized macronutrient distribution, allowed for individualized nutritional therapy, as evidenced by improved weight and serum albumin levels. This significantly enhanced the patient’s quality of life, underscoring the need for heightened awareness of nutritional support’s prognostic importance in oncology patients.^[16]^

The tumor microenvironment (TME) serves as the critical niche for tumor cell survival. Exploring TME dynamics holds significant implications for elucidating the pathogenesis and progression of DLBCL, guiding clinical treatment strategies, and predicting prognostic outcomes. Tumor heterogeneity, coupled with its propensity for recurrence and metastasis, contributes to the generally poor survival rates observed in many malignancies.^[17,18]^ The complex interactions among immune cell subsets within the TME suggest their potential regulatory roles in tumor proliferation, apoptosis, and microenvironmental remodeling.^[19]^ Relapsed/refractory diffuse large B-cell lymphoma (R/R DLBCL) demonstrates poor response to conventional therapies and is associated with short median survival. With advancements in tumor immunotherapy, CAR-T therapy has emerged as a promising modality with demonstrated targeted cytotoxicity against malignant cells. However, precise therapeutic efficacy assessment and individualized treatment adjustments remain crucial. In this case, the patient declined further antitumor therapy and invasive diagnostic procedures following gastrointestinal hemorrhage, thereby precluding the implementation of evidence-based immunotherapeutic regimens (including monotherapy/combination immune checkpoint inhibitors or CAR-T cell therapy) per standard clinical protocols.^[20]^ Failed to accurately represent the potential therapeutic benefits of immunotherapy in this patient population.

## 4. Conclusion

For patients with DLBCL exhibiting extranodal involvement complicated by TEF, treatment strategies should be maximally individualized through multimodal therapy integrating chemotherapy, radiotherapy, and immunotherapy to address primary disease. Regarding TEF fistula management, early nutritional support following diagnosis represents an effective non-surgical intervention.

## 5. Study limitations

This study is constrained by its single-case research design, which limits the generalizability of the findings. Furthermore, further follow-up is necessary to evaluate the impact on the patient’s quality of life and the potential occurrence of complications.

## Author contributions

**Conceptualization:** Wei Cao.

**Data curation:** Xunlai Lu, Luyao Ma, Li Ma, Yan Chen, Wei Cao.

**Formal analysis:** Xunlai Lu, Wei Cao.

**Investigation:** Xunlai Lu, Luyao Ma, Li Ma, Yan Chen, Wei Cao.

**Methodology:** Xunlai Lu, Luyao Ma, Li Ma, Yan Chen, Yuanjia Luo, Shengfu Liu, Wei Cao.

**Project administration:** Xunlai Lu, Wei Cao.

**Supervision:** Wei Cao.

**Validation:** Xunlai Lu, Wei Cao.

**Writing – original draft:** Xunlai Lu, Wei Cao.

**Writing – review & editing:** Xunlai Lu, Luyao Ma, Li Ma, Yan Chen, Yuanjia Luo, Shengfu Liu, Wei Cao.
